# Queuing Theory to Decrease Waiting Times in Emergency Department

**DOI:** 10.5812/atr.10473

**Published:** 2014-03-15

**Authors:** Fernando-Miguel Cantero, Maximino Redondo

**Affiliations:** 1 Research Network for Health Services in Chronic Disease (REDISSEC), Costa del Sol Hospital, University of Malaga, Marbella, Spain

**Keywords:** Queuing Theory, Emergencies

Dear Editor,

Regarding the article: Application of queuing theory to decrease waiting times in emergency department: Does it make sense? published in Archives of Trauma Research (1), I would like to make some comments. Although results of the study by Alavi-Moghaddam et al. using queuing theory are particularly interesting to analyze the impact of a new strategy to reduce waiting times before implementing it, all the proposals offered an increase of resources or staff: adding one or more senior emergency residents on each shift, adding one more bed to intensive care unit, adding another clerk to take electrocardiograms in ED, and expanding the laboratory staff and specialist consultants by 50%. It has been argued that adding resources is not simply sufficient to fix the flow problems in ED (2). Although there are a variety of medical, social, financial and other external causes for crowding, the internal organization of ED is often a source of inefficiencies. Dr. Ng et al. in an ED from Ontario (3), attempted various solutions to improve waiting times. They increased the number of triage nurses and stations, added medical directives, increased physician staffing, hired a nurse practitioner and opened a fast track area. Despite increasing resources and staffing, there was little appreciable impact on overall waiting times; therefore they tried another way to improve ED efficiency. They applied philosophies and tools from the Toyota Production System (Lean thinking) to improve productivity and reduce waiting times. By eliminating waste from their internal ED processes, improving workplace organization, focusing on reducing interruptions and internal waits, and continuously refining improvements, waiting times, length of stay, and patient satisfaction improved with no additional staff or beds. The goal of Lean is to refine production in such a way that work flows smoothly from one step to the next with no wasted time, effort, or resources. The essential elements of each step are identified. Any step that does not add value to the product is considered waste or muda. The process is then reorganized to eliminate any muda. The new process is then standardized, mistake-proofed, and implemented, a process of continual, incremental improvement called Kaizen in Japanese. R. Holden critically reviewed 18 articles describing the implementation of Lean in 15 ED in the United States, Australia and Canada (4). The review revealed numerous ED process changes, often involving separate patient streams, structural changes such as new technologies, communication systems, staffing changes, and the reorganization of physical space. Patient care usually improved after implementation of Lean, with decreases in the length of stay, waiting times, and proportion of patients leaving the ED without being noticed. Success factors included employee involvement, management support, and preparedness for change. However, Lean is not a panacea, but rather a tool that may or may not succeed, according to the efforts surrounding its use. Dickinson et al. showed the first case series with negative results in a hospital that had attempted to implement Lean (5). What was common across the successful EDs was the strict adherence to Lean principles.
